# Cultivating Professional Identity Formation in Medical Students: a Mixed-Methods Study of Nutrition-Focused Service Learning

**DOI:** 10.1007/s40670-026-02757-w

**Published:** 2026-05-13

**Authors:** Asia T. Bright, Noor Rejjal, Caitlin McAfee

**Affiliations:** 1 McGovern Medical School at the University of Texas Health Science Center at Houston, 6431 Fannin Street Suite G.150, Houston, TX 77030 USA; 2 Assessment and Evaluation McGovern Medical School at the University of Texas Health Science Center at Houston, 6431 Fannin Street Suite G.150, Houston, TX 77030 USA

**Keywords:** Professional identity formation, Nutrition education, Service learning, Undergraduate medical education, Language Inquiry Word Count (LIWC)

## Abstract

Professional identity formation (PIF) is a central goal of medical education, shaping how future physicians integrate clinical responsibility and health equity. Nutrition is fundamental to these professional commitments, yet nutrition education is often marginalized and disconnected from identity formation. This pilot study examined a community garden–based nutrition service-learning curriculum as a site for socially mediated PIF. Thirty-one first-year medical students completed a validated PIF Scale pre-intervention and reflective writings post-intervention analyzed using Language Inquiry Word Count-22. Findings demonstrate alignment between PIF domains and linguistic markers of reflection, social engagement, and prosocial values, suggesting experiential nutrition education supports identity development.

## Background

Professional identity formation (PIF) is increasingly recognized as a core outcome of undergraduate medical education, referring to the developmental process through which learners internalize the values, roles, and norms of the medical profession [1]. Cruess et al.’s framework conceptualizes PIF as a dynamic, socially mediated process shaped by formal curricula, informal learning experiences, role modeling, and reflective practice [2].

Within this framework, nutrition represents a foundational yet frequently marginalized component of professional formation, despite its significant relevance to patient care, prevention, and health equity [3]. Although nutrition is formally included in medical curricula, instruction is predominantly delivered through didactic modalities that offer limited opportunities for participation, reflection, or identity-relevant meaning making [4]. Reflecting growing national concern regarding this gap, the U.S. House of Representatives passed a bipartisan resolution in 2022 (House Resolution 1118, 117th Congress [2021–2022]) calling for meaningful nutrition education for medical trainees, citing escalating healthcare expenditures related to nutrition-associated disease and substantial federal investment in graduate medical education through Medicare [3]. This policy context underscores the importance of integrating nutrition into medical education in ways that support professional responsibility and identity development, rather than knowledge acquisition alone.

Experiential and service-learning pedagogies align closely with Cruess et al.’s emphasis on socialization, participation in situated learning, social participation, and reflection as mechanisms of identity development [5]. Community-based garden service learning offers a context in which medical students can engage with nutrition not merely as abstract knowledge, but as a socially embedded practice linked to community health, responsibility, and professional values. Such settings allow students to enact aspects of the physician role (e.g., advocate, educator, collaborator) and supports identity formation through participation and reflection rather than knowledge acquisition alone.

Despite growing recognition of PIF as a central educational outcome, methodological challenges persist in its assessment. Consistent with Cruess et al.’s emphasis on reflection and narrative as windows into identity development, PIF and service-learning research has traditionally relied on qualitative reflective writing [6]. In contrast, medical education research more broadly privileges quantitative, psychometrically validated measures [6].

### Current Study

To address this divide, we used a mixed-methods approach that integrates both quantitative and qualitative perspectives on professional identity formation (PIF).

In the current study, PIF was assessed quantitatively using the validated Tagawa PIF scale [7], and qualitatively after the intervention through reflective writing analyzed using Linguistic Inquiry and Word Count (LIWC), a computational linguistic tool that allows systematic examination of identity-relevant language features [8].

By examining the relationship between quantitative PIF measures and linguistic patterns within reflective narratives, this study explore whether computational analysis of reflective writing can serve as a theoretically grounded and methodologically rigorous complement to traditional assessment approaches. More broadly, this study aims to advance efforts to operationalize PIF in a manner that respects its complex, socially situated, and narrative character, while remaining compatible with the quantitative conventions of medical education. Ethical approval for this study was obtained from the Institutional Review Board.

### Activity

As a prerequisite to the service-learning curriculum, students completed the Tagawa PIF scale to assess baseline professional identity development [7]. In this pilot study, 31 first-year medical students were divided into three small groups and assigned to two community gardens that served as partner sites for the required service-learning experience. At each site, students met with urban garden leaders to learn about community-identified needs, local food systems, and the relationship between nutrition and health. Students then engaged in hands-on garden activities, including soil preparation, weeding, and planting. In addition, students walked through surrounding communities directly impacted by the garden initiatives. Following completion of the service-learning experience, students returned to the medical school and responded to a structured reflective prompt, capturing their experiences and perceived impact on their development as future physicians.

PIF was assessed using the Tagawa PIF Scale administered prior to participation in the service-learning intervention [7]. Participants completed the 15-item Tagawa PIF Scale, with responses on a 7-point Likert scale ranging from 1 (“completely inapplicable”) to 7 (“greatly applicable”), and a neutral midpoint of 4. Several items were reversely coded per the original validation study (Cronach alpha = 0.72) to ensure consistent interpretation of professional identity attributes. There are five theoretically informed subscales: (1) self-control as a professional, (2) awareness of being a medical doctor, (3) reflection as a medical doctor, (4) execution of social responsibility, and (5) external and internal self-harmonization. Higher scores indicate greater alignment with characteristics of mature professional identity. Descriptive statistics were calculated for total scores, and internal consistency reliability was assessed using Cronbach’s alpha (α = 0.548).

Following completion of the service-learning experience, participants submitted written reflections describing their experiences and perceived impact on their development as future physicians. Reflective narratives were analyzed using Linguistic Inquiry and Word Count (LIWC-22), a validated computerized text analysis tool that quantifies psychologically and socially meaningful word categories [8]. Selection of LIWC domains was guided a priori by the subdomains of the Tagawa survey (self -control, awareness of being a medical doctor, reflection as a medical doctor, execution of social responsibility, external and internal self harmonization) and that composite LIWC scores were constructed to approximate those same constructs. This mapping was theory-driven rather than one-to-one, reflecting the complexity of translating narrative constructs into linguistic indicatorsLIWC-22 analyzes text by calculating the proportion of words that fall into predefined psychological and linguistic categories (e.g., insight, affect, social processes). For example, a reflective statement such as ‘I realized how my role extends beyond clinical care into the community’ would contribute to LIWC categories including insight (e.g., ‘realized’), self-referential language (e.g., ‘I’), and social processes (e.g., ‘community’). Higher proportions in these categories indicate greater relative use of such language within a given reflection. This approach allows for systematic quantification of narrative features that are theoretically aligned with professional identity formation constructs.

To examine convergence between psychometrically assessed PIF and linguistically expressed reflective processes, correlation analyses were conducted within Tagawa PIF subscale scores and their corresponding LIWC domain. This approach was intended to assess whether a service-learning garden experience functions as an observable manifestation of identity-related processes described in Cruess et al.’s framework through language use in reflective narratives. Statistical significance was set at *p* < .05. All analyses were conducted using SPSS version 31.

## Results

Consistent with widely accepted best-practice threshold in LIWC-based text analysis, reflective essays containing fewer than 100 words were excluded from analysis (*n* = 6) to improve the stability and interpretability of linguistic estimates.

Due to differences in measurement modality and score scaling between pre- and post-intervention measures, descriptive comparisons focus on distributional patterns rather than direct equivalence of raw means. Z-scores were calculated separately for pre-intervention Tagawa scores and post-intervention LIWC variables using their respective distributions. As such, differences in mean z-scores reflect relative positioning within each distribution rather than direct comparability across time points.

As shown in Table 1, standardized descriptive statistics revealed a consistent post-intervention pattern across all professionalism-related constructs. Pre-intervention Tagawa self-assessment scores demonstrated negative mean *z* values with limited variability, whereas post-intervention LIWC-derived reflection scores showed positive mean *z* values accompanied by substantially greater dispersion.

**Table 1:** Standardized (Z-Score) descriptive statistics for pre- and post-intervention measures

Measure	Group	M	SD	*N*	Cohen’s d
Self-Harmonization	Pre (Tagawa)	−0.16	0.36	31	0.36
	Post (LIWC)	0.20	1.42	25	
Execution / Social Responsibility	Pre (Tagawa)	−0.17	0.17	31	0.37
	Post (LIWC)	0.22	1.46	25	
Reflection as a Medical Doctor	Pre (Tagawa)	−0.17	0.28	31	0.36
	Post (LIWC)	0.21	1.44	25	
Positive Self-Control	Pre (Tagawa)	−0.17	0.23	31	0.36
	Post (LIWC)	0.21	1.45	25	
Awareness of Being a Medical Doctor	Pre (Tagawa)	−0.17	0.22	31	0.36
	Post (LIWC)	0.21	1.45	25	

Z-scores were calculated within each measure as (score − sample mean)/SD; accordingly, pre- and post-intervention values reflect relative standing within their respective distributions and should not be interpreted as a common metric of pre–post change. Cohen’s d represents the standardized mean difference between pre-intervention Tagawa scores and post-intervention LIWC-based scores and is presented for descriptive purposes only, not as a direct estimate of longitudinal change

Standardized mean differences indicated small-to-moderate effects across all professionalism-related constructs (Cohen’s *d* ≈ 0.36), with consistently higher post-intervention scores following the gardening intervention. Although effect sizes were modest in magnitude, their consistency across domains and alignment with increased post-intervention variability suggest meaningful educational impact on reflective and professional identity expression.

As shown in Table 2, correlation analyses revealed distinct patterns between pre- and post-intervention measures. Pre-intervention Tagawa self-assessment scores demonstrated generally weak to moderate associations, with only a limited number of significant relationships among execution, self-control, and professional awareness. In contrast, post-intervention LIWC-derived reflection scores showed strong to very strong positive correlations across all constructs (*r*s = 0.40–0.99, *ps* < 0.05), indicating substantial relatedness following the gardening intervention. Although this pattern may reflect integration of professionalism, self-regulation, and professional identity within learners’ narratives, these high coefficients should be interpreted cautiously, as they may also indicate overlap among LIWC-derived constructs, shared linguistic features, or analytic redundancy.

**Table 2:** Pearson correlations among standardized professionalism constructs by time point

Pre-Intervention (Tagawa PIF; *n* = 31)					
Variable	1	2	3	4	5
1. Self-Harmonization	—				
2. Execution / Social Responsibility	−.02	—			
3. Reflection as a Medical Doctor	.03	.15	—		
4. Positive Self-Control	−.30	.44*	.10	—	
5. Awareness of Being a Medical Doctor	−.25	.38*	.17	.42*	—
Post-Intervention (LIWC Reflection; *n* = 25)					
Variable	1	2	3	4	5
1. Self-Harmonization	—				
2. Execution / Social Responsibility	.44*	—			
3. Reflection as a Medical Doctor	.99**	.40*	—		
4. Positive Self-Control	.97**	.41*	.99**	—	
5. Awareness of Being a Medical Doctor	.45*	.98**	.41*	.41*	—

Values are Pearson correlation coefficients (*r*). All variables are standardized (*z* scores). *p* < .05. ** *p* < .01 (two-tailed)

## Discussion

This pilot study suggests that urban gardening service-learning is associated with patterns of reflective expression consistent with professional identity formation processes in first-year medical students. Using a mixed-methods design combining validated self-report measures with computational linguistic analysis of reflective writing, the study offers preliminary evidence that experiential, nutrition-focused learning environments may support PIF.

Pre-intervention Tagawa PIF scores showed negative mean z values within limited variability, whereas post-intervention LIWC-derived scores demonstrated positive mean z values and greater dispersion. This pattern aligns with PIF theory emphasizing individualized reflection within authentic professional contexts [2]. Because pre-intervention PIF and post-intervention reflective language were assessed using different measurement modalities, findings are interpreted as differences in patterns of expression rather than direct within-subject change.

Exploratory standardized mean differences (Cohen’s d ≈ 0.36) were calculated to describe magnitude of distributional differences; however, because these comparisons involve non-equivalent measures, effect sizes should be interpreted descriptively rather than as direct intervention effects.

Correlation analyses further highlighted change: baseline PIF constructs showed weak associations, whereas post-intervention LIWC domains were strongly intercorrelated.

Although this pattern may suggest integration of self-regulation, professional awareness, social responsibility, and reflection within students’ narratives, these high coefficients should be interpreted cautiously. Given the LIWC-based and narrative-derived nature of the post-intervention variables, the observed correlations may also reflect overlap among linguistic categories, shared lexical features, or partial analytic redundancy across related constructs. Accordingly, the findings support substantial convergence among reflective domains, but not necessarily their full conceptual distinctiveness.The uniform directionality of effects across domains, coupled with greater post-intervention variability, supports the interpretation that the observed pattern reflects a meaningful educational signal rather than random fluctuation .

## Limitations

This study has several important limitations that should be considered when interpreting the findings. First, the sample size was relatively small, which may limit statistical power and the generalizability of results. Additionally, the exclusion of reflections containing fewer than 100 words may have introduced bias by disproportionately removing less detailed or less expressive responses, potentially skewing the linguistic analysis toward participants who were more reflective or verbose. However, this is a commonly adopted best-practice threshold in LIWC-based text analysis, employed to enhance the reliability and interpretability of derived linguistic measures [9].

Second, the study design relied on non-equivalent measures across time points, with pre-intervention professional identity assessed the Tagawa PIF scale and post-intervention outcomes derived from LIWC analysis of reflective writing. This limits the ability to draw conclusions about changes in professional identity over time and necessitates cautious interpretation of differences observed across modalities.

Third, the internal consistency of the Tagawa PIF scale in this sample was moderate, and lower than that reported in the original study, raising concerns about the reliability of the pre-intervention measure and the stability of baseline estimates. Please note that this estimate was derived from a relatively small sample (*n* = 31), which may have contributed to instability in the reliability coefficient [10].

Finally, the very high correlations observed among post-intervention LIWC variables warrant careful interpretation. While these may reflect conceptual integration of professional identity constructs, they may also indicate overlap among linguistic categories or analytic redundancy inherent in LIWC-derived measures [8]. Taken together, these limitations suggest that findings should be viewed as preliminary and hypothesis-generating, and future research using larger samples, consistent measurement approaches, and refined analytic strategies is needed to further validate and extend these results.

## Conclusions

Collectively, these findings indicate that community-based nutrition service-learning may promote integration of professional identity in ways not captured by pre-intervention self-report alone. This study is the first to apply LIWC to reflective writing in medical education, providing preliminary and exploratory, data ofofhow computational linguistic analysis can render narrative expressions of PIF empirically visible. Consistent with Cruess et al.’s conceptualization of professional identity formation, this approach captures nuanced, language-based indicators of the developmental processes underlying identity formation. This study provides a proof-of-concept for integrating computational linguistic analysis with professional identity formation research in medical education. It demonstrates the potential of using tools such as LIWC to systematically capture and quantify reflective, identity-related processes in learner narratives.Furthermore, it offers a novel method for measuring the trajectory of professional identity as a developmental journey, complementing traditional self-report instruments. Despite its small sample size, this pilot provides evidence that even a single experiential learning activity can yield reflective patterns of professional identity formation.
